# Cyberbullying Victimization and Non-suicidal Self-Injurious Behavior Among Chinese Adolescents: School Engagement as a Mediator and Sensation Seeking as a Moderator

**DOI:** 10.3389/fpsyg.2020.572521

**Published:** 2020-11-05

**Authors:** Chengfu Yu, Qi Xie, Shanyan Lin, Yue Liang, Guodong Wang, Yangang Nie, Jianping Wang, Claudio Longobardi

**Affiliations:** ^1^Department of Psychology and Research Center of Adolescent Psychology and Behavior, School of Education, Guangzhou University, Guangzhou, China; ^2^Department of Psychology, University of Turin, Turin, Italy; ^3^Human Resources Department, Guangzhou University, Guangzhou, China; ^4^School of Politics and Public Administration, South China Normal University, Guangzhou, China

**Keywords:** cyberbullying, school engagement, sensation seeking, adolescent, non-suicidal self-injury

## Abstract

Although a large body of research has indicated that cyberbullying victimization is a crucial risk factor for adolescent non-suicidal self-injury (NSSI) behavior, the mediating and moderating mechanisms underlying this relationship remain unclear. To address this research gap, this study, based on the social control theory and the organism-environment interaction model, was designed to test whether school engagement mediated the relationship between cyberbullying victimization and adolescent NSSI and whether this mediating effect was moderated by sensation seeking. A sample of 1,102 adolescents (*M*_age_ = 13.17; *SD* = 0.69) anonymously completed the questionnaires. The results showed that the positive association between cyberbullying victimization and adolescent NSSI was mediated by school engagement. Moreover, this indirect link was significant for adolescents with high-level sensation seeking but non-significant for adolescents with low-level sensation seeking. These findings highlight school engagement as a potential mechanism linking cyberbullying victimization to adolescent NSSI, and high sensation seeking was an important risk factor to amplify this indirect effect. Intervention programs aimed at reducing NSSI among adolescents may benefit from the current research.

## Introduction

Non-suicidal self-injury (NSSI) behavior refers to the deliberate, direct, and socially unacceptable destruction of body tissue in the absence of suicidal intent, such as skin cutting, skin burning, hitting oneself, and so on (Nock, 2010). NSSI has become a serious global public health problem. According to the results of survey research, The lifetime prevalence of NSSI among adolescents worldwide is 17.2% (Swannell et al., 2014), the 12 months prevalence of adolescent NSSI in China was relatively high, ranging from 15 to 32.7% (Jiang et al., 2017; Tang et al., 2018; Siu, 2019). Liu et al. (2020) use a sample of 2,716 Chinese adolescents found that the prevalence of NSSI has reached 26.9% in the past 12 months. Furthermore, the developmental consequences of NSSI during adolescence impact a wide array of aspects well-being later in life, and the effects can be far-reaching. These consequences can include anxiety, depression, and future suicidal behaviors (You and Lin, 2015; Davico et al., 2019). Therefore, it is a pressing need to identify the factors that may have important implications for adolescents’ NSSI. The current research tend to explore school engagement as mediator and sensation seeking as moderator in the relationship of cyberbullying victimization and adolescent NSSI. Potential findings of such research may provide insights for developing effective intervention and prevention programs to reduce the prevalence of NSSI.

### Cyberbullying Victimization and Adolescent NSSI

Victimization is a risk factor for adolescents’ NSSI (Claes et al., 2015; Baiden et al., 2017). The interpersonal model of NSSI suggests that individuals experiencing negative interpersonal events usually may use NSSI as a maladaptive coping strategy to relieve stress or tension to escape from painful and stressful experiences (Nock, 2010). With the rapidly growing population of Internet users, cyberbullying victimization is becoming increasingly common among adolescents. Cyberbullying refers to one individual or group of individuals who repeatedly communicate hostile or aggressive messages intended to inflict harm or discomfort on others (e.g., usually peers) by a set of behaviors performed through the network of connections (e.g., computers, smartphones) (Li, 2007; Smith et al., 2008; Chan and Wong, 2015). The reported prevalence of cyberbullying victimization varies, with estimates ranging from 15 to 35% for Chinese adolescents (Erreygers et al., 2018; Li et al., 2019). Results from several empirical studies suggest that cyberbullying victimization was positively associated with NSSI and suicidal behavior (Van Geel et al., 2015; Moore et al., 2017; John et al., 2018). Further empirical studies’ findings indicate that adolescents who are bullied (including cybervictimization) are more likely to engage in NSSI (Baiden et al., 2017) and that cyberbullying victimization is associated with a higher risk of suicidality among teenagers (Messias et al., 2014). These findings highlight cyberbullying victimization as a potentially important risk factor for adolescent NSSI. Therefore, the current research explore the mechanism of NSSI affected by risk factors (e.g., cyberbullying victimization), so as to further understand the possible causes of NSSI in adolescents and provide theoretical reference for the prevention and reduction of adolescent NSSI in the future.

### School Engagement as a Potential Mediator

A majority of adolescents’ social interactions and learning activities take place in their schools (Sujung and Min, 2018). Hirschi’s social control theory (Hirschi, 1969) states that if individuals have experienced a lack of social bonds (e.g., low school engagement), they were incline to develop delinquent or problematic behaviors. According to the Social Control Theory (Hirschi, 1969), experiencing victimization, such as cyberbullying victimization, may reduce a student’s level of school engagement, which may, in turn, further influence their problematic behaviors, such as engaging in NSSI. This relationship between school engagement and victimization suggests that school engagement may mediate the impact of cyberbullying victimization on adolescent NSSI.

School engagement is a multifaceted construct that incorporates students’ “initiation of action, effort, and persistence on schoolwork, as well as ambient emotional states during learning activities” (Skinner et al., 1990; p. 24). The possible mediating effects of school engagement are suggested by the following facts: First, adolescents who experience cyberbullying victimization are less likely to feel bonded to school and/or engaged in school activities (Buhs et al., 2006; Li et al., 2020). This disengagement occurs because the cyberbullied students tend to have lower levels of psychological resources (including psychological security, self-esteem, self-efficacy, and so on), which can, in turn, reduce their initiative for engaging in school activities (Na et al., 2015). In a longitudinal study, Buhs et al. (2006) found that middle school students who are bullied and who continually experience victimization are at higher risk for school disengagement. Similarly, Li et al. (2020) reported that bullying victimization could significantly reduce middle school students’ emotional and cognitive school engagement.

Additionally, when less engaged in school activities, adolescents are more likely to develop NSSI (Young et al., 2011). The school environment plays a significant role in shaping adolescents’ behaviors, and adolescents may be more inclined to participate in risk-taking behaviors (including NSSI behaviors) when they are less engaged in school (Pittman and Richmond, 2007). For example, Wyman et al. (2010) found that improving students’ school engagement can effectively and significantly reduce their suicide-risk level. Considerable evidence has confirmed that school engagement is an important protective factor against NSSI (Chapman et al., 2011; Kim et al., 2019). Chapman et al. (2011) found that emotional school engagement was associated with lower injurious behavior, including NSSI behavior in adolescents. Moreover, Kim et al. (2019) reported that emotional school engagement could buffer the negative impact of cybervictimization on adolescent NSSI and suicidal behaviors. Based on the literature reviewed above, school engagement may be a crucial mediator in the underlying mechanism that how cyberbullying victimization brings a bear to adolescent NSSI, so we propose the following:

Hypothesis 1: school engagement will mediate the relationship between cyberbullying victimization and adolescents’ NSSI.

### Sensation Seeking as a Moderator

Despite what is known about the significant role of cyberbullying victimization in adolescent NSSI, not all adolescents are equally influenced by cyberbullying victimization (Moore et al., 2017). Hence, there must be some potential moderators that buffer or aggravate the risk effect of cyberbullying victimization on adolescent NSSI. According to the organism-environment interaction model (Cummings et al., 2002), individual behavior (e.g., NSSI behavior) is formed and developed in the interaction between the individual and the environment. Namely, when adolescents in the interaction of different levels of intrapersonal attributes (e.g., sensation seeking) and environmental contexts (e.g., cyber, school), they would respond differently to their developmental outcomes (e.g., NSSI behavior). Sensation seeking is a form of difficult temperament that refers to “the seeking of varied, novel, complex and intense sensations and experiences” (Zuckerman et al., 1972). According to the organism-environment interaction model perspective (Cummings et al., 2002), cyberbullying victimization may influence adolescent’s NSSI in conjunction with sensation seeking. Sensation seeking has been identified by numerous empirical researchers to be a robust risk factor for emotional and behavioral problems (Knorr et al., 2013; Laurence et al., 2015). However, to the best of our knowledge, no study to date has tested the moderating effect of sensation seeking on direct or mediating pathways from cyberbullying victimization to adolescent NSSI and other maladjustments. However, some researchers have confirmed that high sensation seeking and impulsivity exacerbate the risk effect of stress on adolescent self-injurious thoughts and behaviors (Pierro et al., 2012; You and Leung, 2012; Aldrich et al., 2018). For example, in a 6-months longitudinal study, Aldrich et al. (2018) found that high impulsivity increased adolescents’ self-injurious thoughts and behaviors that result from stress-induced low psychological arousal.

Additionally, some empirical research results have confirmed that sensation seeking amplifies the risk effect of school adversity on adolescent risk behaviors. For instance, Eklund and Fritzell (2014) found that the interaction of sensation-seeking with negative school effects was associated with an increased risk of adolescents’ delinquent behaviors. Moreover, some empirical research has confirmed that high sensation seeking could significantly aggravate the detrimental effects of the consequences of low school engagement (e.g., substance abuse) on adolescent self-injurious thoughts and behaviors (Ortin et al., 2012). For instance, Ortin et al. (2012) found that sensation seeking significantly amplifies the adverse effect of adolescents’ substance use problems on their suicide attempts. Based on the above theoretical framework and empirical evidence, we propose the following:

Hypothesis 2: sensation seeking will moderate the positive indirect link between cyberbullying victimization and adolescent NSSI. Specifically, this indirect link will be significant among adolescents with high sensation seeking but less significant among adolescents with low sensation seeking.

### The Present Study

**FIGURE 1:**
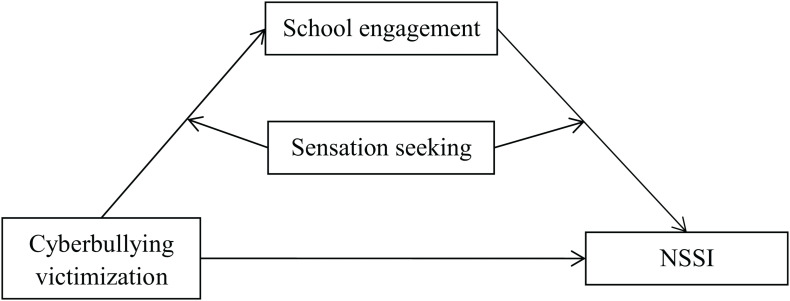
The proposed mediated moderation model. NSSI, non-suicidal self-injurious behavior.

In the current study, we aimed to bring together the social control theory (Hirschi, 1969) and the organism-environment interaction model (Cummings et al., 2002) to explain why cyberbullying victimization is associated with adolescent NSSI. We aimed to produce a moderated mediation model based on the combined effects described by hypothesis 1 and hypothesis 2 (see Figure 1).

## Materials and Methods

### Participants

Participants were recruited from three junior middle schools in Guangdong province, southern China, through stratified and random cluster sampling. A total of 1,006 adolescents (51.78% females, *n* = 521) ranging in age from 12 to 15 (*M*_age_ = 13.16, *SD* = 0.67) participated in this study. There were 556 seventh graders and 450 eighth graders. Reflecting the demographics of the sample, 48.63% of participants’ fathers and 54.07% of their mothers have less than a high school education. 44.51% come from rural areas, and 55.49% from cities.

### Measures

#### Cyberbullying Victimization

Cyberbullying victimization was measured with the Cyberbullying Victimization Scale (Erdur-Baker, 2007). Participating adolescents were asked to report the frequency of cyberbullying victimization they experienced while communicating through the Internet in the past 6 months. The scale provides statements to which participants respond using a 4-point Likert-type score ranging from 1 = never happened to 4 = more than five times. An example of the survey statements is, “Someone once spread untrue and bad things about me on the Internet.” Average scores were calculated for all items in this scale, with higher scores indicating a higher frequency of cyberbullying victimization. This scale has been shown adaptive reliability and validity among Chinese adolescents in previous studies (Zhou et al., 2013; Chu et al., 2018). The result of confirmatory factor analysis (CFA) indicates the scale has good structure validity in this study: χ^2^/*df* = 5.01, CFI = 0.88, RMSEA = 0.06, and SRMR = 0.05. Moreover, in this study, Cronbach’s α for this scale was 0.82.

#### NSSI

NSSI was measured with the 7-item Non-Suicidal Self-Injury Scale (You et al., 2013). Adolescents were asked to report whether they have engaged in NSSI behaviors (i.e., self-cutting, burning, scratching skin and so on) in the past six months. Items were rated on a 4-point Likert scale (1 = *never*, 2 = *once or twice*, 3 = *three to five times*, 4 = *six times or more*). The result of CFA indicates the scale has very good structure validity in this study: χ^2^/*df* = 4.54, CFI = 0.98, RMSEA = 0.06, and SRMR = 0.02. In the current study, the Cronbach’s α coefficient of this scale was 0.71.

#### School Engagement

A 23-item self-report school engagement questionnaire was used to assess school engagement (Wang et al., 2011). The measure of school engagement has three dimensions, including emotional engagement (e.g., “*I feel happy and safe in this school*”), behavioral engagement (e.g., “*How often do you have trouble paying attention in classes?*”), and cognitive engagement (e.g., *“How often do you try to figure out problems and planning how to solve them?*”). Items were rated on a Likert scale with 5 points, ranging from 1 = “*never*” to 5 = “*always*” for emotional engagement and behavioral engagement, and from 1 = “*fully disagree*” to 5 = “*fully agree*” for emotional engagement. Average scores of all items were calculated on this scale, with higher scores representing the higher levels of school engagement. The result of CFA indicates the scale has very good structure validity in this study: χ^2^/*df* = 4.08, CFI = 0.93, RMSEA = 0.06, and SRMR = 0.05. The scale of school engagement has adequate reliability and validity for Chinese adolescents, in which was proved by previous study (Zhu et al., 2015). In this study, the Cronbach’s α coefficient of this questionnaire was 0.89.

#### Sensation Seeking

The sensation-seeking subscale of the UPPS-P Impulsive Behavior Scale (Cyders et al., 2014) was used to assess sensation seeking. This scale has been shown adaptive reliability and validity among Chinese adolescents in previous studies (Zhu et al., 2016; Wang et al., 2017). The adolescents were asked to answer four items by indicating the level of sensation seeking (e.g., “I sometimes like doing things that are a bit frightening”). Items were rated on a Likert scale with 4 points (ranging from 1 = *strongly disagree* to 4 = *strongly agree*). Average scores were calculated for all items in this scale, and higher composite scores indicate higher levels of sensation seeking. The result of CFA indicates the scale has excellent structure validity in this study: χ^2^/df = 1.62, CFI = 0.99, RMSEA = 0.03, and SRMR = 0.01. In this study, the Cronbach’s α coefficient of this subscale was 0.74.

### Control Variables

Given that parent-adolescent communication is a significant factor influencing adolescent NSSI (Ru et al., 2018), we controlled for this variable in the statistical analyses. The relevant measurement was taken using a parent-adolescent communication questionnaire (Su et al., 2013). Participants indicated how frequently they spoke with their parents regarding daily life, academics, interpersonal interaction, safety, and emotional issues on a 3-point scale ranging from 1 = *never* to 3 = *often*. Average scores of all items were calculated on this scale, with higher scores representing higher levels of parent-adolescent communication. The result of CFA indicates the questionnaire has excellent structure validity in this study: χ^2^/*df* = 3.48, CFI = 0.99, RMSEA = 0.05, and SRMR = 0.02. In this study, the Cronbach’s α coefficient of this questionnaire was 0.91.

### Procedure

This research received ethics approval, with full permission and consent from the Academic Ethics Review Committee of the School of Education, Guangzhou University. Before adolescent participants filled out any of the self-report scales for this study, we received written, signed informed consent from all the adolescent participants, their parents/legal guardians, their teachers, and their schools. In addition, we informed participants that they could quit the research test at any time they wished to. The data were collected by well-trained psychology teachers or undergraduate students who majored in psychology. The data were collected in the format of paper and pencil within the participants in their classrooms. In the process of data collection, the professional staff informed the participants, in advance, that all the collected data are anonymous and would only be used for scientific research purposes. Also, there are no “correct” (right or wrong) answers for any of the choices, allowing participants to respond to the questionnaires according to their true thoughts.

### Statistical Analyses

This study used the SPSS 25.0 software for reliability analysis and descriptive statistical analysis. Moreover, we conducted structural equation modeling using maximum likelihood estimation and bootstrapping with 1,000 replicates to test the mediation and moderation effects in Mplus 7.1 (Muthén and Muthén, 1998–2019). According to statisticians’ suggestion (Hoyle, 2012), we used three indices (including χ^2^/*df*, CFI, and RMSEA) to evaluate the goodness of fit of a model. The model fit is considered acceptable when χ^2^/*df* < 5, CFI > 0.90, RMSEA < 0.08, and SRMR < 0.08 (Hoyle, 2012).

## Results

### Preliminary Analyses

The means, standard deviations, and correlation coefficients for all variables of the current study are displayed in Table 1. The results indicate that cyberbullying victimization and sensation seeking were both negatively correlated with school engagement and positively correlated with NSSI. Moreover, school engagement scores were negatively correlated with NSSI.

**TABLE 1:** Descriptive statistics and correlations for all variables.

Variables	1	2	3	4	5	6	7
1.Gender	1.00						
2.Age	0.06*	1.00					
3.PAC	−0.02	−0.14**	1.00				
4.CV	0.02	0.00	−0.14**	1.00			
5.SS	0.05	−0.06	−0.06	0.14**	1.00		
6.SE	−0.05	0.00	0.41**	−0.16**	−0.16**	1.00	
7.NSSI	−0.03	0.03	−0.18**	0.24**	0.14**	−0.19**	1.00
Range	0–1	11.58–16.17	1–3	1–4	1–4	1–5	1–7
Mean	–	13.16	2.26	1.13	2.00	3.91	1.09
*SD*	–	0.67	0.53	0.20	0.67	0.49	0.29

*p < 0.05, **p < 0.01. Gender and age were dummy coded such that 1 = male, 0 = female. PAC, parent-adolescent communication; CV, Cyberbullying victimization; SS, sensation seeking; SE, school engagement; NSSI, non-suicidal self-injurious behavior.

### Testing for Mediating Effect of School Engagement

**FIGURE 2:**
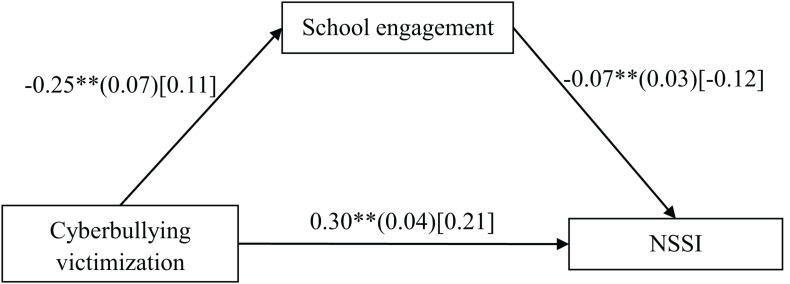
Model of the mediating role of school engagement between cyberbullying victimization and NSSI. NSSI, non-suicidal self-injurious behavior. The values outside the brackets are unstandardized coefficients, those in parentheses are standard errors, and those in brackets are standardized coefficients. Paths between gender, age, parent-adolescent interaction, and each of the variables in the model are not displayed. Of those paths, the following were significant: age (*b* = 0.02, *SE* = 0.02, β = 0.06, *t* = 2.11, *p* < 0.05, 95% CI [0.003, 0.09]), and parent-adolescent interaction (*b* = 0.20, *SE* = 0.01, β = 0.40, *t* = 13.73, *p* < 0.01, 95% CI [0.17, 0.23]) to school engagement; Parent-adolescent interaction to NSSI (*b* = 0.03, *SE* = 0.01, β = −0.09, *t* = −2.82, *p* < 0.01, 95% CI [−0.05, −0.01]). ^∗∗^*p* < 0.01.

The mediation model represented in Figure 2 revealed an excellent fit to the data: χ^2^/*df* = 2.34, CFI = 0.98, RMSEA = 0.04, and SRMR = 0.02. Cyberbullying victimization negatively predicted school engagement (*b* = −0.25, *SE* = 0.07, β = −0.11, *t* = −3.59, *p* < 0.01, 95%CI [−0.39, −0.11]), and school engagement negatively predicted NSSI (*b* = −0.07, *SE* = 0.02, β = −0.12, *t* = −3.59, *p* < 0.01, 95%CI [−0.11, −0.03]). Moreover, the residual effect of cyberbullying victimization on NSSI was significant (*b* = 0.30, *SE* = 0.04, β = 0.21, *t* = 6.86, *p* < 0.01, 95%CI [0.22, 0.39]). Bootstrapping analyses indicated that school engagement significant mediated the relation between cyberbullying victimization and adolescent NSSI (indirect effect = 0.018, *SE* = 0.008, 95% CI [0.005, 0.036]).

### Testing for Moderated Mediation

**FIGURE 3:**
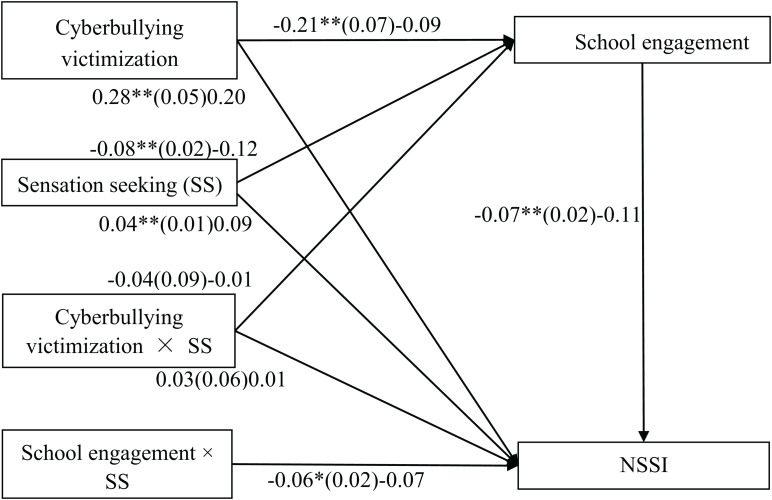
Model of the moderating role of sensation seeking on the indirect relationship between cyberbullying victimization and NSSI. SS, sensation seeking; NSSI, non-suicidal self-injurious behavior. The values outside the brackets are unstandardized coefficients, those in parentheses are standard errors, and those in brackets are standardized coefficients. Paths between gender, age, parent-adolescent interaction, and each of the variables in the model are not displayed. Of those paths, the following were significant: parent-adolescent interaction to school engagement (*b* = 0.19, *SE* = 0.02, β = 0.40, *t* = 13.61, *p* < 0.01, 95% CI [0.17, 0.22]) and NSSI (*b* = −0.03, *SE* = 0.01, β = −0.10, *t* = −3.01, *p* < 0.01, 95% CI [−0.05, −0.01]). ^∗^*p* < 0.05, ^∗∗^*p* < 0.01.

**FIGURE 4:**
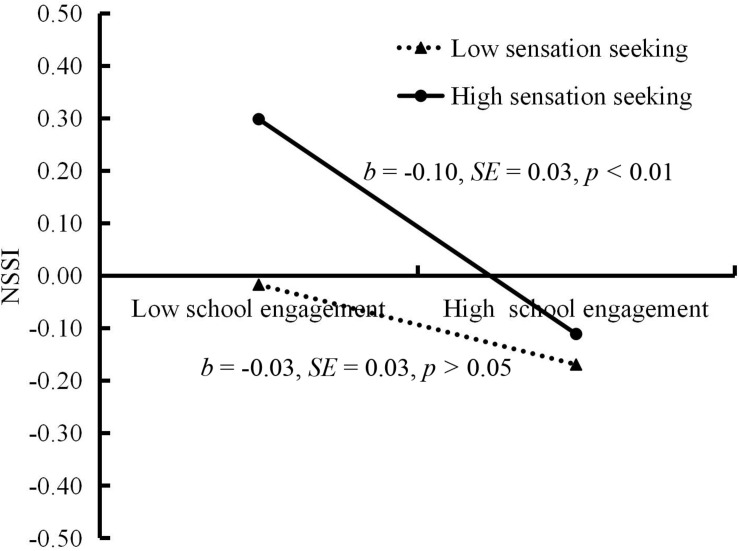
NSSI among adolescents as a function of school engagement and sensation seeking. NSSI, non-suicidal self-injurious behavior.

The moderated mediation model represented in Figure 3 revealed a good fit to the data: χ^2^/*df* = 2.63, CFI = 0.99, RMSEA = 0.04, and SRMR = 0.03. The bias-corrected percentile bootstrap results indicated that the indirect effect of cyberbullying victimization on adolescent NSSI through school engagement was moderated by sensation seeking. Specifically, sensation seeking moderated the association between school engagement and NSSI (*b* = −0.06, *SE* = 0.02, β = −0.07, *t* = −2.35, *p* < 0.05, 95% CI [−0.11, −0.01]). We conducted a simple slopes test, and, as depicted in Figure 4, school engagement was significantly associated with NSSI among the adolescents with higher sensation seeking (1 *SD* above the mean; *b* = −0.10, *SE* = 0.03, *t* = −3.95, *p* < 0.01, 95% CI [−0.16, −0.05]). However, this link between school engagement and NSSI was not significant among the adolescents with lower sensation seeking (1 *SD* below the mean; *b* = −0.03, *SE* = 0.03, *t* = −1.02, *p* > 0.05, 95% CI [−0.08, 0.02]). Moreover, sensation seeking had a significant negative association with school engagement(*b* = −0.08, *SE* = 0.02, β = −0.12, *t* = −3.99, *p* < 0.01, 95% CI [−0.13, −0.04]) and a significant positive relationship with NSSI (*b* = 0.04, *SE* = 0.01, β = 0.09, *t* = 2.86, *p* < 0.01,95% CI [0.01, 0.06]). However, the interaction between cyberbullying victimization and sensation seeking in predicting school engagement and NSSI were not significant.

Moreover, the indirect link between cyberbullying victimization and NSSI via school engagement was significant for the adolescents with higher sensation seeking (indirect effect = 0.025, *SE* = 0.015, 95% CI [0.001, 0.062]). However, this indirect link was non-significant for those with lower sensation seeking (indirect effect = 0.005, *SE* = 0.006, 95% CI [−0.002, 0.029]). Therefore, the mediating effect of school engagement between cyberbullying victimization and adolescent NSSI was moderated by sensation seeking. Furthermore, we conducted a supplementary analysis. The results indicated that no paths were moderated by gender.

## Discussion

Despite burgeoning evidence for the risk effect of bullying(include cyberbullying victimization) on adolescent NSSI (Baiden et al., 2017; Esposito et al., 2019), few researchers have examined the mechanisms involved in mediating and moderating effect. To address this research gap, we tested whether school engagement mediated the relationship between cyberbullying victimization and adolescent NSSI and whether this mediating effect was moderated by sensation seeking.

Consistent with hypothesis 1 and the prediction derived from the social control theory (Hirschi, 1969), the current study found that school engagement mediated the positive association between cyberbullying victimization and adolescent NSSI. In other words, adolescents who experience cyberbullying victimization may reduce their school engagement, which may, in turn, increase their problematic behaviors such as NSSI. Thus, insufficient school engagement is one of the explanatory mechanisms for why adolescents are more likely to adopt NSSI to cope with their experience of cyberbullying victimization. In line with previous studies, adolescents who report being bullied and perceived unsafety and distrust at school (i.e., low school engagement) were more inclined to develop NSSI (Noble et al., 2011). Emotional school engagement also has been found to play a significant indirect role between cyberbullying victimization and suicidal behavior (Kim et al., 2019). Additionally, school engagement may be a crucial protective factor for the effective prevention and reduction of adolescent NSSI; this was also supported by prior studies (Chapman et al., 2011; Kim et al., 2019). For example, Chapman et al. (2011) found that higher levels of emotional school engagement were associated with lower injurious behavior. Previous studies have illustrated there are some mediators (e.g., depressive mood and symptoms) between bullying victimization and adolescent NSSI (Claes et al., 2015; Baiden et al., 2017). The current research further demonstrated the mediating role of school engagement in the relationship between cyberbullying victimization and adolescent NSSI. Therefore, this finding enriches the body of research regarding the potential mechanisms between cyberbullying victimization and adolescent NSSI. Additionally, previous studies pointed out that assessing the influence factors of adolescents’ NSSI behaviors could be help to comprehend the formulation of specific strategies for treating members of this behavior (Ong et al., 2017). This study provides insights that may be useful for developing intervention programs aiming to reduce the incidence of adolescent NSSI. For example, the study found that cyberbullying victimization was a risk factor and school engagement was a exactly protective factor for adolescent NSSI behavior, thus, intervention programs of school could focus on these two empirically validated factors and add effective interventions to reduce adolescent NSSI (Hasking et al., 2016).

Our findings were consistent with hypothesis 2 and the prediction derived from the organism-environment interaction model (Cummings et al., 2002). Our results suggest that the risk effect of cyberbullying victimization on adolescent NSSI via school engagement was significant for adolescents with high-level sensation seeking but non-significant for adolescents with low-level sensation seeking. Specifically, the risk effect of low school engagement on adolescent NSSI was significantly exacerbated by high sensation seeking. This effect probably occurs because adolescents with high sensation-seeking levels are more likely to use maladaptive regulation strategies rashly (e.g., affiliate with deviant peers) when they lack emotional support from teachers and classmates (Zhu et al., 2016), which in turn increases NSSI. This result may also be explained by the reverse-buffering model (Rueger et al., 2016), which proposes that a risk factor strengthens the negative connection between a beneficial factor and a maladjustment outcome. More specifically, high levels of sensation seeking reinforce the negative impacts of low school engagement on adolescents’ NSSI.

This study also reveals that sensation seeking did not moderate the association between cyberbullying victimization and adolescent school engagement and NSSI. Specifically, high sensation seeking cannot exacerbate the adverse direct impacts of cyberbullying victimization on adolescent development. This finding may reflect that cyberbullied adolescents often lack adequate psychological resources and the interpersonal support resources needed to deal with cyberbullying victimization (Na et al., 2015). The lack of skills and support puts them at high risk for school disengagement and NSSI, regardless of the protective effects of low sensation seeking. It may also suggest that cyberbullying victimization has a comparatively robust impact on adolescents’ school engagement and NSSI, and sensation seeking may only modulate cyberbullying victimization’s psychological and behavioral effects (e.g., school engagement) on NSSI. Further explorations of other crucial moderating variables (such as school connectedness, Kim et al., 2019) are necessary to determine which factors could aggravate or buffer the impacts of cyberbullying victimization.

In conclusion, the creative point of this study is to produce reliable data to construct a moderated mediation model for exploring “how and when” the potential risk and protective factors take effect on adolescents’ NSSI. Specifically speaking, cyberbullying victimization has negative impact on adolescents’NSSI via school engagement when adolescents with high-level sensation seeking. We found that school engagement was a protective factor for NSSI among adolescents with low sensation seeking. However, among adolescents with high sensation seeking, school engagement cannot withstand the detrimental impact of being cyberbullied on their NSSI. These results emphasize the significant role of school engagement and sensation seeking in the relationship between cyberbullying victimization and NSSI among Chinese adolescents. Thus, the current study offers an analysis model to recognize the role of school aspect (school engagement) and personal factor (sensation seeking) between cybervictimization and NSSI, which is an important contribution to advance in the understanding of adolesceent cyberbullying phenomena and its negative impact on their NSSI behavior. Meanwhile, the current study provide some reference value for future related research and prevention programs developed for Chinese adolescent NSSI.

## Limitations and Future Directions

Undoubtedly, the current research has some limitations, but it also provides some possible directions for future research. First, all the assessments in this study were reported by the adolescents; thus, common method bias might exist to a certain degree (Du et al., 2005). Second, this research was performed using a cross-sectional study design, so the results cannot uncover the causal relationships between studied variables (Maxwell et al., 2011). Longitudinal tracking designs can be considered in the future. Third, this research only explored the mediating role of school engagement and the moderating role of sensation seeking in the relationship between cyberbullying victimization and NSSI among Chinese adolescents, which undermines the cross-cultural generalizability of the conclusions. Future research may consider other mediating variables (e.g., basic psychological needs satisfaction, Emery et al., 2017) and moderating variables (e.g., attachment with peers and parents, Jiang et al., 2017) to further explore the mechanisms between cyberbullying victimization and adolescents’ NSSI in eastern and western cultures.

## General Conclusion

1. The positive association between cyberbullying victimization and adolescent NSSI was mediated by school engagement.
2. This indirect link of cyberbullying victimization on adolescent NSSI via school engagement was significant for adolescents with high-level sensation seeking but non-significant for adolescents with low-level sensation seeking.

## Data Availability Statement

The raw data supporting the conclusions of this article will be made available by the corresponding authors, without undue reservation, to any qualified researcher.

## Ethics Statement

The studies involving human participants were reviewed and approved by the Academic Ethics Review Committee of the School of Education, Guangzhou University. Written informed consent to participate in this study was provided by the participants’ legal guardian/next of kin.

## Author Contributions

CY, QX, SL, YN, and JW conceived and designed the research. CY and JW performed the research. CY analyzed the data. CY, QX, SL, YL, GW, YN, JW, and CL contributed to the writing of the manuscript, revised the manuscript critically for important intellectual content, and commented on and approved the final manuscript. All authors contributed to the article and approved the submitted version.

## Conflict of Interest

The authors declare that the research was conducted in the absence of any commercial or financial relationships that could be construed as a potential conflict of interest.
